# Effect of crude extracts of *Moringa stenopetala* and *Artemisia absinthium* on parasitaemia of mice infected with *Trypanosoma congolense*

**DOI:** 10.1186/1756-0500-7-390

**Published:** 2014-06-24

**Authors:** Tsegabirhan Kifleyohannes, Getachew Terefe, Yacob H Tolossa, Mirutse Giday, Nigatu Kebede

**Affiliations:** 1 College of Veterinary Medicine, Mekele University, Mekele P. O. Box: 231, Ethiopia; 2 School of Veterinary Medicine, Addis Ababa University, Debre Zeit P. O. Box 34, Ethiopia; 3 Aklilu Lemma Institute of Pathobiology, Addis Ababa University, Addis Ababa P. O. Box 1176, Ethiopia

**Keywords:** *Artemisia absinthium*, *Moringa stenopetala*, Crude extract, Mice, Parasitaemia, *Trypanosoma congolense*

## Abstract

**Background:**

Treatment of trypanosomosis is currently facing a number of problems including toxicity of trypanocidal drugs and development of resistance by the parasites. These limitations have prompted the search for alternative active substances (such as of natural origin). The purpose of the study was to investigate the effect of extracts of *Moringa stenopetala* and *Artemisia absinthium* on *Trypanosoma congolense in* mice.

**Methods:**

Swiss white male mice aged 8–12 weeks were divided into six experimental groups of six animals. Water and methanol extracts of the two plants were prepared. *T. congolense* was isolated from cattle at Ghibe valley (Ethiopia). All experimental mice received approximately 1 x 10^5^ trypanosomes in 0.2 ml of blood. Plant extracts were given orally to four groups (2 plant species and two extraction methods) at 400 mg/kg body weight for seven consecutive days. One group remained as distilled water treated control and the other as diminzene aceturate treated control. The effect of the extracts on levels of parasitaemia, body weight, packed cell volume (PCV) and mice survival was monitored for 25 days.

**Results:**

All treatments have significantly reduced parasitaemia and helped improve body weight, PCV and survival of mice compared to the water-treated control (P < 0.01 in all cases). These effects were comparable to that with diminazene aceturate. No significant difference was observed in the reduction of parasitaemia between plant extract treatment groups. However, mice with extracts of *A. absinthium* had significantly higher body weight than those with extracts of *M. stenopetala* (P < 0.05).

**Conclusions:**

The two plants have antitrypanosomal potential against *T. congolense* by reducing the levels of parasitaemia, maintaining good PCV and body weight, and prolonging the lives of infected animals.

## Background

Among those diseases that have plugged sub-Saharan Africa is the African animal trypanosomosis. In the absence of vaccines, trypanosomosis control relies heavily on vector control and chemotherapy. Targeting the trypanosomes in the host using chemotherapeutic agents is one of control practices undertaken since the advent of modern trypanocidal drugs. Unfortunately the use of these trypanocides is beset by numerous limitations, including toxicity of the drugs, development of resistance by the parasites [1,2] and limited availability (cost and accessibility). Investigations in different parts of Ethiopia indicate the widespread occurrence of trypanocidal resistance problems [3-5].

These limitations of commercially available trypanocidal drugs have prompted the search for alternative active substances (such as of natural origin). Works on plant extracts active against some species of trypanosomes have already shown promising findings in many countries including Ethiopia. Examples are extracts of *Neurolaena lobata* on *Trypanosoma cruzi*[6], *Moringa stenopetala* on *T. b. brucei*[7], *Combertum molle* on *T. b. rhodesiense*[8] and *Artemisia spp*. on *T. brucei*[9]. However, information is scant on the efficacy of these and others plants on some trypanosome species of major livestock threat. Therefore, this study was undertaken with the objective of investigating the effects of crude extracts of *Moringa stenopetala* and *Artemisia absinthium,* in *Trypanosoma congolense* infected mice.

## Results

### Toxicity test in uninfected mice

**Figure 1:**
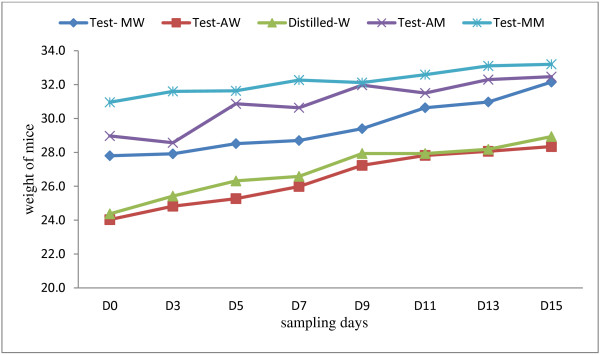
**Weights of uninfected mice during subacute toxicity testing.** The mice were given either water and methanol extracts of *A. absinthium* and *M. stenopetala* or water only (MW = *M. stenopetala* in water, AW = *A. absinthium* in water, Distilled-W = water, AM- *A. absinthium* in methanol*,* MM- *M. stenopetala* in methanol).

**Figure 2:**
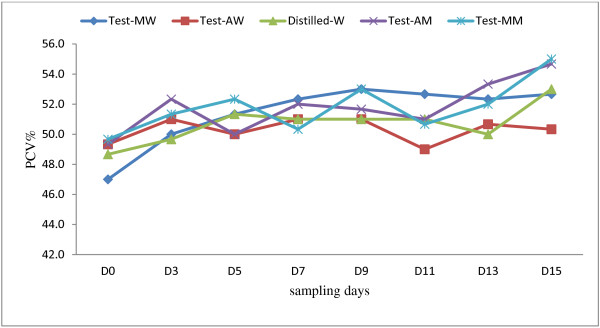
**PCVs of uninfected mice during subacute toxicity testing.** The uninfected mice were treated with either water and methanol extracts of *A. absinthium* and *M. stenopetala* or water only (MW = *M. stenopetala* in water*,* AW = *A. absinthium* in water, Distilled-W = water, AM- *A. absinthium* in methanol*,* MM- *M. stenopetala* in methanol).

Different extracts and doses of *A. absinthium* and *M. stenopetala*, given orally did not significantly affect the levels of packed cell volume and weight of the uninfected mice throughout the experimental period when compared to those of water- treated mice. All the mice showed a gradual increase in body weight (Figure 1 and PCV (Figure 2) during the toxicity test. Moreover, no sign of acute toxicity was detected.

### Parasitaemia in trypanosome infected mice

**Figure 3:**
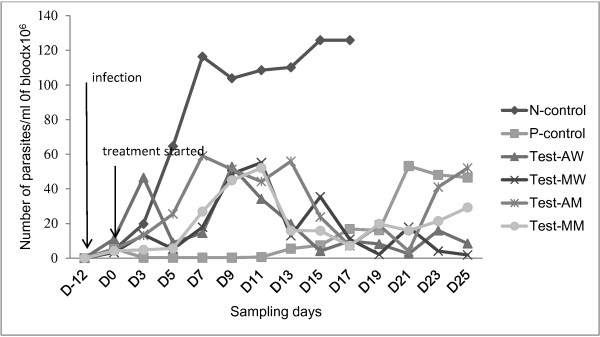
**Parasitaemia with *T. congolense *infection in mice treated with both water and methanol extract of *M. stenopetala*, *A. absinthium, *diminazene aceturate or water only (N = negative, P = positive, AW =** ***A. absinthium *in water, MW =** ***M. stenopetala *in water, AM =** ***A. absinthium *in methanol, MM =** ***M. stenopetala *in methanol).**

In N-control group of mice, peak parasitaemia was observed on D7 post treatment where mean counts reached 11.6×10^7^/ml. This was followed by a higher count on D15 and afterwards. Mice treated with single dose of diminazene aceturate showed non-detectable parasitaemia three days post treatment until D11. This was followed by a gradual increase in parasite count until D19. In animals treated with water and methanol extracts of *A. absinthium* and *M. stenopetala*, peak parasitaemias were observed on Days 7 and 11 post treatment but parasite counts where approximately half that of the non-treated control group (Figure 3). All treatments at 95 % CI (AW mean: 1.8×10^6^ ± 1.6×10^6^, CI: 0.2×10^6^-3.4×10^6^; MW mean: 1.7×10^6^ ± 1.8×10^6^, CI: 0.9×10^6^-2.2×10^6^; AM mean: 2.3×10^6^ ± 2.0×10^6^, CI: 0.3×10^6^-4.3×10^6^; MM mean: 2.0×10^6^ ± 1.5×10^6^, CI: 0.5×10^6^-3.5×10^6^) have significantly reduced the parasitaemia of mice compared to the water treated control (8.6×10^6^ ± 4.6×106, CI: 3.0×10^6^- 11.6×10^6^), (P < 0.01) while the effect of all plant extracts in the reduction of parasitaemia was comparable to that of diminazene aceturate (P > 0.05). There was no significant difference in the reduction of parasitaemia between crude extract treatment groups.

### Impact of treatment on the weight of trypanosome-infected mice

**Figure 4:**
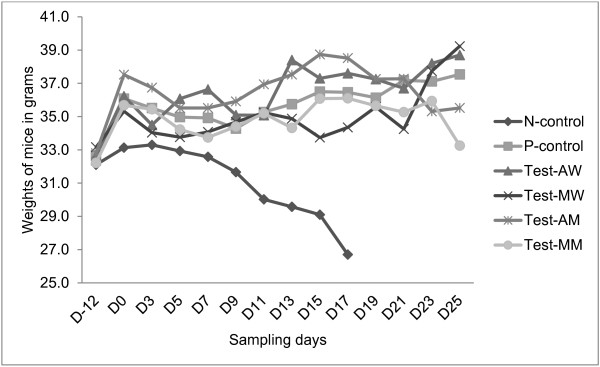
**Weights of *T. congolense *** **infected mice treated with both water and methanol extract of *M. stenopetala*, *A. absinthium, *** **diminazene aceturate or water only.**

The weight in the untreated infected mice group started to decrease after 12 days post infection and continues to decrease till all the mice died by D18. In the diminazene aceturate treated group, after a slight decline on D9, the animals showed a gradual increase in weight until the end of the experiment. Water extract of *A. absinthium* and *M. stenopetala* treated mice groups generally showed a gradual increase in mean weight until the end of the experimental period. After some decrease around D7 and D9, groups treated with methanol extract of *A. absinthium* and *M. stenopetala* showed a gradual increase in body weight. Groups treated with plant extracts and diminazene aceturate showed significantly higher body weights than the water treated control group (P < 0.01) (Figure 4). The effect of all plant extracts on body weights of infected mice was comparable to that of diminazene aceturate (P > 0.05). However, group receiving water extract of *A. absinthium* had significantly higher body weight (mean: 35.9 ± 0.3 g, CI: 35.1-36.7 g) than water extract of *M. stenopetala* (mean: 34.7 ± 0.4 g, CI: 33.7-35.7 g) (P < 0.05). Similarly, methanol extract of *A. absinthium* had significantly higher body weight (mean: 36.5 ± 0.3 g, CI: 35.8-37.1 g) than methanol extract of *M. stenopetala* (mean: 34.8 ± 0.3 g, CI: 34.1-35.4 g) (P < 0.01).

### Packed cell volume in trypanosome infected mice

**Figure 5:**
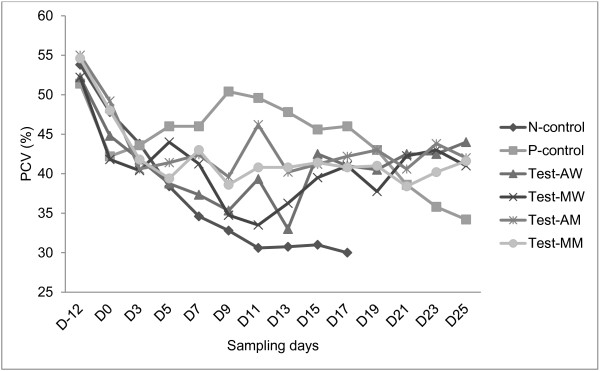
**PCVs of *T. congolense *** **infected mice treated with both water and methanol extract of *M. stenopetala*, *A. absinthium, *** **diminazene aceturate or water only.**

While the mean PCV in the N-control group continued to decrease till all the mice are lost due to the infection, all the other treatment groups generally controlled their PCV to fairly normal level with significantly higher values than the N-control group (P < 0.05) (Figure 5). Except with water extract of *M. stenopetala*, all plant extracts had comparable effect to diminazene aceturate and there was no significant difference in the level of PCV between crude extract treatment groups.

### Survival periods of trypanosome infected mice

**Figure 6:**
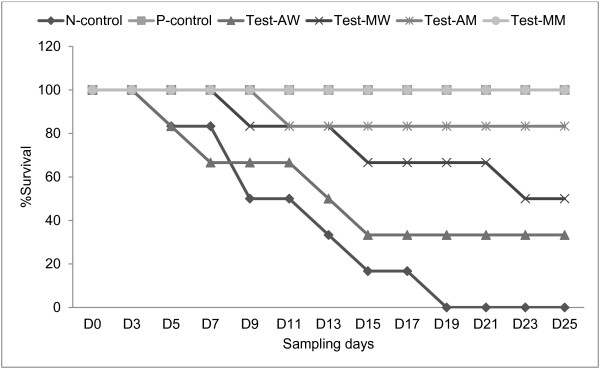
**Effect of water and methanol extracts of *M. stenopetala*, *A. absinthium *** **on *T. congolense *** **infected mice survival time.**

Figure 6 shows survival curve of infected mice with or without treatment. Deaths in water-treated mice (N-control) started 5 days after treatment with all the mice dying by 18 days post treatment. All animals have survived in groups treated with diminazene aceturate and methanol extract of *M. stenopetala*. In Water extract of *A. absinthium* treated mice 2 survived while in water extract of *M. stenopetala* treated mice three survived to the end of experimental period. In methanol extract of *A. absinthium* treated mice one animal died on day 9 and the rest survived until the end of the experiment. Diminazene aceturate and methanol extracts of *M. stenopetala* and *A. absinthium* had significantly increased survival time compared to untreated infected mice (p < 0.05).

## Discussion

All animals in the non-treated control group have already died by the 18^th^ day of the experimental treatment suggesting that the *T. congolense* strain isolated from Ghibe has readily established and was highly pathogenic to mice. The *in vivo* antitrypanosomal activity exhibited against *T.congolense* by the crude extracts of the plants *M. stenopetala* and *A. absinthium* are in agreement with the findings of previous workers on other species of trypanosomes such as *T. cruzi*, *T. b. brucei* and *T. b. rhodesiense*[10-12]. However, further study may be needed to identify the exact molecule(s) that has/have played a major role in the killing and/or suppression of *T. congolense* infection in mice. Studies on *M. stenopetala*[7] and *A. absinthium*[9] have shown that extracts of the plants had an effect on the growth inhibition of *T. brucei in vitro*. They suggested that this activity of the extracts might be attributed to the major compound camphor and two other major sesquiterpene lactones, absinthin, artabsin and glucosinolates which are known to occur in the plants.

The findings of this study clearly indicated that all the plant extracts exhibited moderate to high antitrypanosomal activity *in vivo.* Comparisons between two solvents of extraction: water and methanol showed, overall, better activity could be obtained when the two plants were extracted with methanol than with water. The reason may be multi-factorial, but it could be attributed to the type of molecules or plant components extracted by the two solvents [13,14].

Despite the significant reduction in parasitaemia, the plant extracts did not completely clear the parasites. Several researchers made similar observations on reduction in parasitaemia and concluded that high parasite load could mask the efficacy of crude extracts [15,16]. Moreover, the crudeness of the extracts, the dose and the oral route of administration might have also reduced the availability of sufficient active compounds.

Observations with the commercial drug, diminazene aceturate showed an initial decrease in the level of parasitaemia to non-detectable level (wet film), and later on during the experimental period, the relapse of parasitaemia. As the animals were under high dose treatment (28 mg/kg), this finding strongly suggests that the parasite isolate has already developed diminazene aceturate resistance. This observation agrees with the findings of studies conducted elsewhere [17,18] that reported diminazene aceturate resistance in livestock in the Ghibe valley (where our sample was taken). Others [4,19] have also reported relapsing parasitaemia after treatment with diminazene aceturate during experimental studies in mice.

The positive effect of plant extracts can further be deduced from weight measurements of the experimental animals. Animals treated with the plant extracts, on average, maintained their body weight at comparable levels to pre-treatment values while those in the untreated infected group showed progressive reduction in body weights. This findings support the reports of similar studies elsewhere [20]. Moreover, animals treated with extracts of the two plants generally had fairly comparable weights to those treated with diminazene aceturate indicating that the reduction in parasitaemia due to the extracts has led to the maintenance of close to normal body weights throughout the experimental period.

It has also been shown that the measurement of anemia gives a reliable indication of the disease status and productive performance of trypanosome-infected animals [21]. Other reports [16,22] have also ascribed acute anemia in trypanosomosis to proliferating parasites. The result of this study showed that these plant extracts have comparable potential to diminazene aceturate since they are able to control anemia, especially at the later stages of the infection, by minimizing drops in PCV values. In untreated mice, the parasite count increased and the packed cell volume (PCV) decreased markedly until the animal dies, which was also observed in previous studies [23,11].

Treatment with plant extracts have shown extended survival of mice in the treatment groups compared with the non-treated control group. The prolongation of survival time in infected groups following oral administration of crude extract agrees with previous reports [15,23] who have applied extracts of *Momordica balsamina* and *Hymenocardia acida* on *T. brucei*. Of special interest are the methanolic extracts of our test plants*,* which clearly demonstrated an interesting antitrypanosomal profile; they maintained the animals above 25 days (with 100% and 83.3% survival rate) despite the presence of parasites in the circulation.

## Conclusion

This study was able to demonstrate the antitrypanosomal effect of crude extracts of *Artemisia absinthium* and *Moringa stenopetala* in *T. congolense* infected Swiss white mice. The findings showed that methanol and water extracts of the leaves of *M. stenopetala*, and aerial parts of *A. absinthium* have suppressive effect on parasitaemia and consequently improved the body weight, PCV and survival of infected mice. The effects of the extracts were comparable to that produced by the commercial drug diminazene aceturate. Hence, this antitrypanosomal potential of the two plants should be further investigated in trypanosomes of livestock importance.

## Methods

### Parasites

This study was conducted on *Trypanosoma congolense* isolates collected from cattle near Ghibe river valley, 176 km south west of Addis Ababa. The altitude of Ghibe ranges from 1,000 to 1,100 m.a.s.l. this site was chosen because of the dominance of the area by *T. congolense* followed by *T. vivax*[24] Blood samples were collected from the ear vein of cattle and examined using wet smear technique to detect the presence of trypanosomes. Suspected *T. congolense* (by motility and morphology) infected blood of cattle were immediately inoculated into five mice, each by 0.2 ml of positive blood intraperitoneally [25] and then transported to the laboratory at Aklilu Lemma Institute of Pathobiology (Addis Ababa University). Further confirmation of the species (*T. congolense*) was done by its establishment in mice and morphological examination after Giemsa staining. The organisms were maintained in the laboratory by serial passage in mice.

### Plant material collection and extraction

Areal part of *A. absinthium* was collected in November 2011 from Sululta area (North of Addis Ababa). Dried and powdered leaves of *M. stenopetala* (originally collected from Arba-Minch area (southern Ethiopia) were obtained from the Forest Research Center (Addis Ababa). The identification of the intact plant materials was done by a botanist at Aklilu Lemma Institute of Pathobiology, Addis Ababa University. One hundred gram of the dried and powdered materials of both plants was soaked separately in 500 ml of distilled water or 80% methanol in a conical flask and stirred intermittently for 72 hrs at room temperature. The materials were then filtered using sterile Whatman No.1 filter paper into a clean conical flask and the residues were re-suspended in the same amount of solvent and then filtered three more times. Pooled aqueous filtrates obtained were freeze dried to powder and stored in a refrigerator at 4°C until used. The pooled methanolic filtrates obtained were evaporated to dryness at 40°C in hot air oven and were re-dissolved in distilled water before oral administration to the animals [15].

### Experimental mice

Swiss white male mice aged between 8–10 weeks and weighing between 30 and 35 grams were obtained from the National Veterinary Institute (Debre-Zeit-Ethiopia), and kept in a fly-proof animal room. They were provided with commercial pelleted ration and water ad-libitum. Thirty-six mice were divided into six experimental groups: N-control (infected but non-treated), P-control (infected and treated with diminazene aceturate), Test-AW (Infected and treated with water extract of *A. absinthium*), Test-MW (Infected and treated with water extract of *M. stenopetala*), Test-AM (Infected and treated with methanol extract of *A. absinthium*) and Test-MM (Infected and treated with methanol extract of *M. stenopetala*).

### In vivo toxicity test

Toxicity test was done on three mice for each plant extract as described in Ngure *et al*. [11]. Mice were orally drenched with a single dose of 2000 mg/kg of extract for acute toxicity followed by another group given 400 mg/kg (experimental test dose) for seven days for subacute toxicity test.

### Experimental infection and treatment

All experimental mice received 1 × 10^5^ parasites in 0.2 ml blood/PBS solution intraperitonially [24]. Development of parasitaemia was followed using the ‘rapid matching’ method [26] on wet films. Most animals showed detectable parasitaemia around day 12 post infection (approximate level of parasitaemia: 5 × 10^6^/ml of blood). Hence, treatments commenced at about day 12 post infection (taken as Day 0) at the first detection of parasitaemia. Crude plant extracts were given at a dose of 400 mg/kg orally for 7 consecutive days while single dose diminazene (28 mg/kg) was given intraperitonially [27]. The infected but non-treated control group received distilled water orally for seven days.

### Measurements

The level of parasitaemia was followed for 25 days post treatment by the ‘rapid matching’ method on blood drawn from the tail every other day. Briefly, a wet film was prepared, covered with a cover slip and slides were examined under 40× objective lens and parasites were counted. The number of fields counted depends on the abundance of parasites per field. If no parasite was counted in 20 fields, then the sample was taken as negative. The number of parasites per milliliter of blood was calculated as described by Herbert and Lumsden [26]. Blood was obtained from the tail vein of mice in heparinised capillary tubes every other day for 12 days before and 25 days after treatment. The tubes were centrifuged in a micro-centrifuge for 5 min at 10000 rpm. PCV was measured by using a haematocrit reader. Similarly, the weight of mice was measured using a weighing balance. The survival rate was measured by recording the number of days the mice has lived with or without treatment, from the day of treatment to 25 days after treatment. On the 25^th^ day post treatment, the percentage of mice surviving was calculated for each group.

### Statistical analysis

Data on parasitaemia, body weight and packed cell volume were analyzed using Windows SPSS Version 15. The one-way ANOVA was used to compare results among and within groups for differences between initial and final results. All data were analyzed at a 95% confidence interval (α = 0.05). Univariate survival analysis of data using Kaplan-Meier method was done to determine the effect of plant extracts on the survival rate of infected animals. The log-rank test was used to examine the null hypothesis that the survival times were identical.

### Ethical considerations

This study was reviewed and approved by the Institutional Review Board (IRB) of the School of Veterinary Medicine, Addis Ababa University. All animals were handled by respecting standard protocols in accordance with the Good Laboratory Practice regulations of EEC Directive of 1986; 86/609/EEC [28].

## Competing interests

The authors declare that they have no competing interests.

## Authors’ contributions

TK: Prepared the first draft of the proposal, collected samples, conducted the experiment, analyzed the data and wrote the first draft of the manuscript. GT: come up with research idea, read and polished the proposal, supervised the conduct of the laboratory experiments, and participated in manuscript write-up. YHT: Read and polished the proposal, supervised the conduct of the laboratory experiments, and participated in the write-up of the manuscript. MG: Read and improved the proposal, identified plant samples, supervised laboratory experiments and read and participated in the write up of the manuscript. NK: participated in the development of the proposal and writ-up of the manuscript. All authors read and approved the final manuscript.
